# Spontaneous Velocity Effect of Musical Expression on Self-Paced Walking

**DOI:** 10.1371/journal.pone.0154414

**Published:** 2016-05-11

**Authors:** Jeska Buhmann, Frank Desmet, Bart Moens, Edith Van Dyck, Marc Leman

**Affiliations:** Institute for Psychoacoustics and Electronic Music, Department of Musicology, Ghent University, Ghent, Belgium; National Institute of Genomic Medicine, MEXICO

## Abstract

The expressive features of music can influence the velocity of walking. So far, studies used instructed (and intended) synchronization. But is this velocity effect still present with non-instructed (spontaneous) synchronization? To figure that out, participants were instructed to walk in their own comfort tempo on an indoor track, first in silence and then with tempo-matched music. We compared velocities of silence and music conditions. The results show that some music has an activating influence, increasing velocity and motivation, while other music has a relaxing influence, decreasing velocity and motivation. The influence of musical expression on the velocity of self-paced walking can be predicted with a regression model using only three sonic features explaining 56% of the variance. Phase-coherence between footfall and beat did not contribute to the velocity effect, due to its implied fixed pacing. The findings suggest that the velocity effect depends on vigor entrainment that influences both stride length and pacing. Our findings are relevant for preventing injuries, for gait improvement in walking rehabilitation, and for improving performance in sports activities.

## Introduction

Studies based on instructed (and intended) synchronization of human locomotion with music show that the expressive features of music can influence the locomotion. Overall, positive outcomes of instructed synchronization with music over no music have been reported. Measurements were related to psychophysical outcomes, physiological outcomes, and kinematic outcomes:

- *Psychophysical outcomes*: Positive results have been found for elite triathletes running on a treadmill while listening to synchronous music [1]. Time-to-exhaustion (TTE) increased by over a minute for both running to motivational and neutral music, compared to the no-music condition. These increases respectively represent an 18.1% and 19.7% improvement in performance. Effects on perceived exertion were small in this maximum intensity test. However, a study by Bood et al. [2] on sub-maximal intensities, did find significant effects on ratings of perceived exertion (RPE).
- *Physiological outcomes*: Oxygen consumption is reported to be lower for athletes running with music compared to running without acoustic stimuli [1]. The use of music is therefore associated with better running economy. Hoffmann et al. [3] mention a decrease in energy consumption to be a result of locomotor-respiratory coupling. When instructed to synchronize cycling to a metronome at the participants’ preferred cycling tempo the coupling increased, and as a result energy consumption decreased. Another effect on physiology was found in the heart rate. The use of music during treadmill running resulted in higher heart rates, specifically at (near-) maximal perceived exertion [2]. This suggests, that music helps runners to work at a higher intensity.
- *Kinematic outcomes*: Beat synchronized walking or running has been studied in terms of the kinematic parameters velocity, stride length, and cadence [4, 5]. If people walked in synchrony with a musical beat, a difference in walking velocity could only be realized through differences in stride length. The stride length being the result of the amount of vigor put into the leg movement, i.e. the embodiment of expressive patterns in the music. Styns et al. [4] examined the effect of music (over metronome) on step length, on a range of different tempi. The study revealed a resonance effect in the walking cadence at approximately 130 beats per minute (BPM) [6, 7]. Based on that result, Leman et al. [5] instructed participants to synchronize their walking pace to musical excerpts with different expression, all at a tempo of 130 BPM. The study showed that some music had activating qualities, increasing the walking velocity, while other music had relaxing qualities, decreasing the velocity compared to the velocity of walking to a metronome.

In this paper, we focus on the effect of tempo-matched expressive music on the velocity and motivation of non-instructed self-paced walking. We call this: the velocity effect. Our research question is twofold.

First, can expressive features of music influence the velocity of self-paced walking, spontaneously? Instructed synchronization implies that humans are asked to adapt to the musical rhythm, irrespective of their preferred (comfort) tempo. However, this imposes a task that can influence the overall velocity effect. In ecological situations we want to work with self-paced walking. Several studies indeed show that self-paced activity is beneficial for motivation and adherence [8–10].

Second, is the velocity of self-paced walking influenced by phase-coherence? Tempo-matched music implies that once the music starts playing, the tempo (and phase) of the music remains fixed. Under this condition, however, it may happen that the human subject entrains to the beat and establishes a stable timing interval between footfall and beat [11]. When that situation happens, we call it phase-coherence, which means that the pace is fixed. Given the fact that velocity depends on both stride length and pace, the question is whether phase-coherence (where pace is fixed) contributes to the velocity effect.

Based on [5] we assume that some music will have an activating influence while other music will have a relaxing influence. These influences will be reflected in the walking velocity. We also assume that this velocity effect can be predicted using acoustical features that capture the musical expressions. Furthermore, we hypothesize that phase-coherence may diminish the velocity effect, because the pace is fixed and therefore the velocity only depends on stride length. When phase-coherence does not occur, velocity may depend on both stride length and pace effects, giving more freedom to adapt the velocity.

To go one step further towards an individualized approach, this study also explores the relation between motivational ratings and kinematic outcome. We hypothesize that when music results in an increased velocity, music will be experienced as more motivational. When music results in an decreased velocity, music will be experienced as less motivational.

## Materials and Methods

### Ethics statement

Participants were informed in advance about the task, the procedure and the technology used for measurement. They had the opportunity to ask questions and were informed that they could finish the experiment at any time. They agreed that recorded movement data would be used for scientific and educational purposes only. In agreement with the general standards at our university and our faculty, security was guaranteed (our indoor task is not dangerous), and privacy is respected. Participants did not have to provide informed consent to participate in this study, because this study only involved behavioral knowledge (cf. 7 May 2004 Belgian Law concerning experiments on the human person (Ch.II, Art.2, Par.11)) and the data were analyzed anonymously. This study as well as the consent procedure was approved by the ethics committee of the Faculty of Arts and Philosophy of Ghent University.

### Participants

The present study included 30 participants (nine male), with an average age of 36.57 years (*SD* = 14.94), ranging from 17 to 77 years old. Average body weight and body length were respectively 67.80 kg (*SD* = 10.10) and 1.70 m (*SD* = 0.08). All participants were physically healthy, 66.67% had some sort of musical education and 33.33% reported using music while walking or jogging. After the experiment all participants received a CD-voucher as a reward.

### Experimental procedure

The experiment took place on a professional 200 m indoor running track in the Flanders Sports Arena of Ghent. Upon arrival, participants were briefly informed about the procedure, after which they were equipped with gait detection sensors and headphones. Participants had to walk 30 min and were instructed to walk as if they were going on a 30 min walk outside. They were not asked to synchronize to the music that would be played to them. To prevent fatigue influencing the results, the 30 min walk was split-up in two blocks of 15 min. This was confirmed afterwards by comparing the first and second part of each block: no significant differences were revealed for stride length with a Friedman’s ANOVA, *χ*^2^ = 2.50, *p* = .48. Furthermore, a repeated measures ANOVA showed no significant differences for stride velocity, *F*(3,87) = 0.40, *p* = .75. During the pause (approximately 15 min), participants could have some water and fruit. Each walking block started with a one-minute warm-up, in which no music was played to the participant. In the following 14 min, a sequence of 15 s of silence and one minute of music was played to the participant. A 15 s period of silence was inserted between songs to reset the walking behavior from the preceding music stimulus. The use of this 15 s silence to prevent a memory effect was checked post hoc by comparing the normalized stride length of four groups of data: relaxing songs preceded by relaxing songs (RR), activating songs preceded by relaxing songs (RA), activating songs preceded by activating songs (AA), and relaxing songs that were preceded by activating songs (AR). A repeated measures ANOVA revealed a significant main effect, *F*(3, 75) = 8.35, *p* < .001. Bonferroni post hoc tests were used to follow up this finding. Significant differences were found between activating and relaxing songs, regardless of the preceding music type: RR (*M* = 100.01%, *SE* = 0.13) vs. RA (*M* = 100.51%, *SE* = 0.13), *p* = .03; RR vs. AA (*M* = 100.62%, *SE* = 0.11), *p* = .002; RA vs. AR (*M* = 100.10%, *SE* = 0.12), *p* = .02; AA vs. AR, *p* = .01. However, RA was not significantly different from AA, *p* = 1.00. Likewise, we did not find significant differences between RR and AR, *p* = 1.00. These results confirm that the preceding song has no memory effect on the stride length during the current song.

During each 15 s of silence DJogger [11] measured the walking cadence of the participant. DJogger then selected a one-minute song with a BPM value within a 5% range of the walking cadence, and adjusted the tempo of that song to exactly match the measured walking cadence, after which the tempo of the music was fixed for the duration of the song. This implicated a song-duration of less than a minute when the tempo was increased, and a song-duration of more than a minute when the tempo was decreased. Depending on the total length of the played songs a participant heard 11 or 12 songs within each 15 min block of walking.

After the experiment, participants were asked to rate the music they had just heard. More precisely, they were asked to indicate how motivating the songs were in the walking exercise. The Brunel Music Rating Inventory 2 (BMRI-2) [12] was used for rating the music. The test was performed in the sports hall on a laptop with headphones. Normally, a BMRI-2 test is used as a pre-test to select a participant’s most motivating songs. This would, however, have had a severe impact on the time necessary to create the music database and the time needed for the BMRI-2 test itself. Instead, we assessed the motivational characteristics of the songs afterwards, which meant maximally 24 songs were evaluated per participant. Finally the participants filled out a questionnaire on personal, musical and sports background.

### Stimuli

Participants were instructed to walk in their own comfort tempo, which is the tempo that participants prefer. Since walking cadence generally varies from 90 to 140 steps per minute (SPM), our music database needed to cover all these tempi. We also took into account that i) the database needed to be large enough for each participant to hear different pieces of music and ii) small enough for multiple participants to hear the same pieces of music as often as possible. All participants had to listen to 22–24 songs and therefore each range of 10 BPM in the database was filled with approximately 22 songs. The music was selected from a large labeled music database using the BPM values and mood labels. The selected songs could have different rhythms, but in the selection process we made sure that the songs had clearly audible rhythms, simple enough to walk on. As a result, almost all selected songs had a 4/4 meter. Earlier research by Leman et al. [5] has shown that some semantic adjectives describing the music are good indicators of activating or relaxing music. The most activating songs were generally labeled as “aggressive” or “loud”, whereas the most relaxing songs were mainly labeled as “tender” or “soft”. In the current study we examined walking behavior on both activating and relaxing music. Therefore, for a pre-selection of songs for our database, we used mood labels that we believed were close to those of Leman et al. [5], like “rebellious”, “energetic” (activating) and “peaceful”, “melancholy” (relaxing). In total 250 songs were pre-selected and then further processed. The perceived loudness of all songs was normalized and one-minute samples were selected from the songs. In LogicProX the beginning and the end of each sample were edited with a linear 50 ms fade-in and a linear 100 ms fade-out. Beat timing information was extracted using BeatRoot [13] and finally the consistency of the tempo throughout the songs was checked with MATLAB. Song tempi were considered to be consistent if the tempo variance was within a range of five BPM. After the music preparation process, 84 of the 250 songs were kept, uniformly spread over the database with respect to their activating or relaxing character. Since the sequence of songs played to each participant depended on the walking cadence of each participant, the set-up of the database ensured the selected sequence of songs to be balanced. Furthermore, the DJogger software selected songs randomly within a 5% tempo difference from the participant’s walking tempo, providing a randomized selection of songs as well. A list of the stimuli can be found in S1 Table.

### Apparatus

Participants were equipped with a number of sensors and wireless headphones. Two OPAL-sensors were strapped around the lower legs of each participant, above the ankle, facing front. A third OPAL-sensor was strapped around the waist of each participant. Gait data collected with these OPAL sensors was used for analysis. This was done in conjunction with MobilityLab^™^ software [14]. Additionally, two iPods were strapped around the lower legs, just above the ankle, facing outward. The iPods communicated wirelessly with the DJogger software [11]. The tempo of the song was manipulated once by DJogger, in order to adapt the selected song to the exact tempo of the walking. This adaptation was done before the start of the song, and the tempo was then kept stable for the complete duration of the song. DJogger normally assures that the song starts in-phase with the walking so that the participant hears the beat at the moment of a footfall. Unfortunately, during our experiment the wireless connection was not always stable and not all songs started in-phase with the walking. To compensate for this, the first 10 seconds of all trials were disregarded in the data analysis so that phase-entrained, if it occurred, could have happened. A headphone receiver was strapped around the upper arm and headphones were placed on the head. All equipment—computers, a dedicated receiver, an Ethernet router, an Arduino link between DJogger and MobilityLab^™^, and a wireless headphones transmitter—was set up next to the walking track.

### Data-analysis

The data for further analysis in this study was derived from the output of the OPAL sensors. MobilityLab^™^ software uses the recorded data from the accelerometers within the sensors to calculate the stride length and stride velocity for each gait cycle (two steps) [14]. Because MobilityLab^™^ and DJogger were synchronized via Arduino, the average stride length and velocity could be calculated for each song and each 15 s silence. The averaged kinematic data for the songs was analyzed statistically with SPSS 22. All the data used for analysis is present in S1 File.

### Normalized and averaged walking data

A participant’s walking behavior depends on his or her body length and weight. Therefore normalized values were calculated for stride length and velocity. For each participant *p* the average stride length and velocity of a song were divided by the average stride length and velocity respectively of its preceding silence. The silences were assumed to be neutral in terms of activation and relaxation. Thus, the normalized values on each preceding silence equaled 100%. If a song had smaller values (e.g. a slower velocity) than its preceding silence, the normalized value was below 100%. In a final step, the mean normalized stride length and velocity were calculated by averaging the normalized values for song *x* over all participants that actually walked to song *x*.

### Phase coherent walking

In addition to the normalized kinematic data we analyzed differences in beat-step synchrony. Because this is temporal periodic data, directional statistics [15] were used to compare the beat and step positions in time. A relative phase angle *ϕ* was calculated for each step and the inter-beat-interval (IBI) the step fell within. In Eq (1) *S*_*t*_ refers to a step at time *t*. *B*_1_ is the time of the beat that occurred before *S*_*t*_ and *B*_2_ is the time of the first beat after *S*_*t*_.

$$\begin{array}{c} \phi =360*\frac{S_{t}-B_{1}}{B_{2}-B_{1}} \end{array}$$ (1)

If the phase angle stayed stable over time this is referred to as phase coherence [16]. Rather than being interested in strict synchronization with the beat (*ϕ* ≈ 0), the focus was on a stable phase at any angle. A way to measure this is by looking at the resultant vector length or the mean phase coherence, as shown in Eq (2), where *N* is the number of samples and *ϕ* is the relative phase angle for step *S*_*t*_. This is a crucial quantity for the measurement of circular spread in directional statistics. The values can range from 0 to 1. |*R*| reaches the value 1 in case of strict phase locking, whereas |*R*| = 0 when phase angles are randomly spread over time, i.e. a uniform distribution of phases.

$$\begin{array}{c} \left|R\right|=\left|\frac{1}{N}\sum_{S_{t}=0}^{N-1}e^{i\phi_{S_{t}}}\right|=1-CV \end{array}$$ (2)

**Fig 1:**
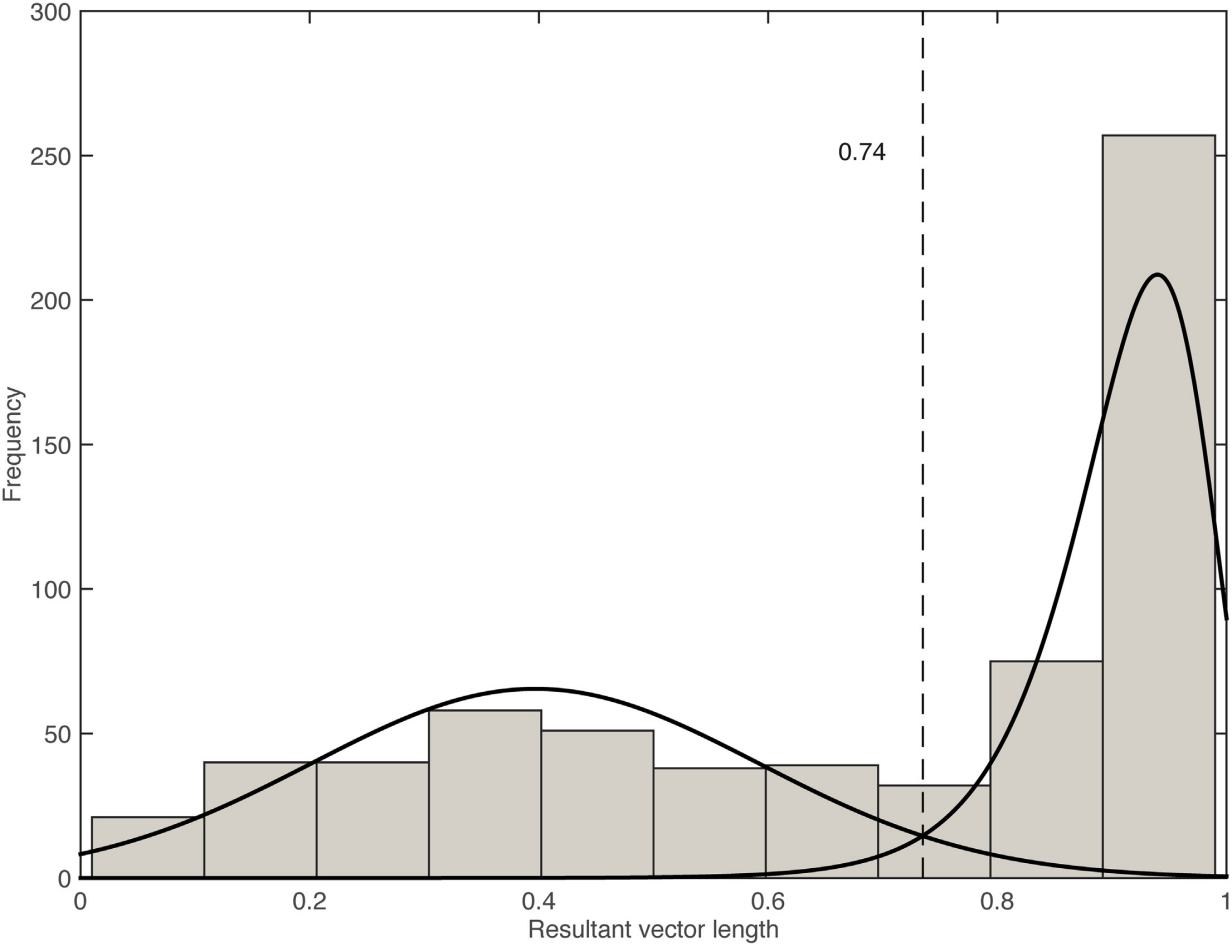
Distribution of resultant vector lengths. This histogram shows |*R*| of all 651 trials, where participants walked to a song, revealing two overlapping processes: phase incoherent walking (low |*R*| values), which is normally distributed, and phase coherent walking, which has an extreme value distribution. The functions estimating those distributions intersect at a value of 0.74.

Fig 1 shows a histogram of all the resultant vector length values. The distribution of these values clearly shows the presence of two overlapping processes: the process of phase incoherent walking and the process of phase coherent walking. The phase incoherent data can be described with a normal distribution (*μ* = 0.40, *σ* = 0.20). The phase coherent data resembles an extreme value distribution.

Given a normal distribution with mean *μ*_*n*_ and standard deviation *σ*_*n*_

$$\begin{array}{c} f\left(x,\mu_{n},\sigma_{n}\right)=\frac{1}{\sqrt{2\pi \sigma_{n}^{2}}}e^{-\frac{{\left(x-\mu_{n}\right)}^{2}}{2\sigma_{n}^{2}}} \end{array}$$ (3)

and an extreme value distribution with mean *μ*_*e*_ and standard deviation *σ*_*e*_, with *x* > 0:

$$\begin{array}{c} F\left(x;\mu_{e},\sigma_{e}\right)=\frac{1}{\sigma_{e}}e^{\left(\frac{x-\mu_{e}}{\sigma_{e}}\right)}e^{-e^{\left(\frac{x-\mu_{e}}{\sigma_{e}}\right)}} \end{array}$$ (4)

then the intersection of both distributions is defined as:

$$\begin{array}{c} \frac{1}{\sqrt{2\pi \sigma_{n}^{2}}}e^{-\frac{{\left(x-\mu_{n}\right)}^{2}}{2\sigma_{n}^{2}}}=\frac{1}{\sigma_{e}}e^{\left(\frac{x-\mu_{e}}{\sigma_{e}}\right)}e^{-e^{\left(\frac{x-\mu_{e}}{\sigma_{e}}\right)}} \end{array}$$ (5)

or

$$\begin{array}{c} \frac{1}{\sqrt{2\pi \sigma_{n}^{2}}}e^{-\frac{{\left(x-\mu_{n}\right)}^{2}}{2\sigma_{n}^{2}}}-\frac{1}{\sigma_{e}}e^{\left(\frac{x-\mu_{e}}{\sigma_{e}}\right)}e^{-e^{\left(\frac{x-\mu_{e}}{\sigma_{e}}\right)}}=0 \end{array}$$ (6)

Using an iterative process this equation can be approached. As a result, the intersection of both functions was found at |*R*| = 0.74. A split in the data at 0.74 resulted in 300 phase incoherent and 351 phase coherent trials. On top of the histogram, Fig 1 shows where the two functions intersect.

### Data partitioning of songs

In order to compare the most relaxing songs to a group of neutral and activating songs, the data set needed to be divided in three groups. The predicted stride lengths for 59 songs were normally distributed. Instead of simply comparing the normalized stride lengths from the ten most activating songs to the ten most relaxing songs and ten songs from the middle of the distribution, we wanted to partition the normal distribution in a statistically sound way. In other words, the aim was to find the threshold limit values (TLV’s) dividing the data in three groups that have an optimal unambiguous character. According to the three-sigma rule of thumb [17] nearly all values of a normal distribution lie within three standard deviations of the mean. In order to keep a sufficient amount of data in all three groups of the distribution, the optimum TLV will be around one standard deviation of the mean, at which approximately 68% of the data will be in the middle—neutral—part of the distribution, and approximately 16% of the data will be considered as the activating group of songs and, equally, 16% of the data as the relaxing group of songs. In total, three multiplication factors of the standard deviation were tested: *m* = 0.8, 1.0, 1.1. For each multiplication factor, three groups of data were derived by using the mean minus *m* ⋅ *σ* and the mean plus *m* ⋅ *σ* as boundaries between the three groups. The middle group contained more songs than the group of relaxing or activating songs; therefore a random selection of approximately the same amount of songs was taken from the middle group. A different random selection was taken 10 times and each time an ANOVA was performed between the actual normalized stride lengths for the three groups of music. The mean and standard deviation were calculated from the *p*-values of the 10 trials. The TLV’s were well chosen once the mean *p*-value was small and stable enough. Table 1 gives an overview of the outcomes at different TLV’s. A multiplication factor of 0.8 was found to define the optimal TLV’s, because the differences between the three groups were found to be the most significant (smallest average *p*-value). On top of that, the data in the three groups were optimally unambiguous, indicated by a very stable *p*-value, i.e. the smallest average standard deviation of the ten *p*-values.

**Table 1:** Division into groups of activating, relaxing and neutral songs, according to different thresholds.

Multiplication factor of *σ*	REL		ACT		NEU	10 ANOVA’s	
	*x* <	#songs	*x* >	#songs	#songs	$\bar{m}$ p-value	$\bar{s}$ p-value
1.0	100.03	10	100.73	10	10	3.02E-05	2.71E-05
1.1	99.99	8	100.76	8	8	5.21E-05	4.84E-05
0.8	100.10	13	100.66	18	16	1.20E-08	7.77E-09

## Results

An analysis of the data is based on three parts. In the first part we analyse the kinematic data from the viewpoint of semantic classification. In the second part, we analyse the kinematic data from the viewpoint of audio-feature classification. In the third part, we consider the relation between kinematics and motivation.

### Semantic classification

In order to obtain a music database with songs that have either an activating or relaxing character, we started from mood labels to select the songs from a large labeled music database. This was based on an association of activating and relaxing characteristics with known semantic labels (see [5]). Songs with mood labels “rebellious”, “bright”, “energetic”, “stimulating” or “happy” were expected to have an activating character. Songs labeled “peaceful”, “melancholy” or “romantic” were expected to have a relaxing effect on walking. However, we observed that a small amount of the mood labels obtained by means of semantic association (e.g., “passionate”, “emotional”, “mysterious”) were ambiguous with respect to their expected activating or relaxing character. For example, some songs associated with the label “emotional” sounded rather relaxing, whereas others sounded more activating. Nevertheless, despite the ambiguity of these labels, we decided to keep those songs in the database because the model that we are aiming at should be able to deal with this kind of ambiguity. After all, the goal of our research is to predict the (activating/relaxing) effect of a song on kinematic parameters from acoustic features, rather than from semantic labels. The semantic labels were only used to create a dataset that is sufficiently relevant for the kinematic effects that we predict.

However, it is of interest to make a comparison between the semantic labels and the actual kinematic response to the music, to check the relevance of our semantic labels post hoc. For this comparison in particular the ambiguous labels were left out.

A paired samples *t*-test was used to compare the kinematic differences. This test revealed that people walked significantly faster to music labeled as activating music (*M* = 100.64%, *SE* = 0.15) than to music labeled as relaxing music (*M* = 99.88%, *SE* = 0.14), *t*(29) = −4.33, *p* < .001, *r* = .63. A similar difference was found for stride length, where strides were larger for activating music (*M* = 100.54%, *SE* = 0.10) than for relaxing music(*M* = 100.08%, *SE* = 0.10), *t*(29) = −3.60, *p* = .001, *r* = .56.

A more in depth analysis was done by splitting the data in phase coherent and phase incoherent trials. Differences in walking behavior between activating and relaxing music were again checked with paired samples *t*-tests. On average, when people were not walking phase coherently with the musical beat, they walked significantly faster on music labeled as activating music (*M* = 100.96%, *SE* = 0.28) than to music labeled as relaxing music (*M* = 99.87%, *SE* = 0.26), *t*(19) = −3.94, *p* < .001, *r* = .67. The normalized stride length was also bigger for music labeled as activating (*M* = 100.66%, *SE* = 0.11), than for music labeled as relaxing (*M* = 100.12%, *SE* = 0.14), *t*(19) = −2.80, *p* = .01, *r* = .54.

However, when people were walking phase coherently with the musical beat, no significant differences in walking velocity between music labeled as activating (*M* = 100.34%, *SE* = 0.12) or relaxing (*M* = 100.13%, *SE* = 0.15) were found, *t*(26) = −1.23, *p* = .23, *r* = .24. This was also the case for stride length: music labeled as activating (*M* = 100.41%, *SE* = 0.13) versus music labeled as relaxing (*M* = 100.13%, *SE* = 0.14), *t*(26) = −1.67, *p* = .11, *r* = .31.

### Audio-feature classification

The goal of this analysis is twofold: (i) can we separate songs in significantly different groups according to kinematic responses? and (ii) can we identify sonic features that are capable of predicting the kinematic response to a song? To answer those questions, we use the same approach of Leman et al. [5] and Varewyck et al. [18]. As a first step, the audio feature extraction algorithm resulted in 185 energy and pitch related features per song. As a second step a regression model was trained (using a cross-validation method) to predict one output per song: a normalized stride length. Finally, a statistical analysis was performed: after dividing the songs in three groups (activating, neutral, and relaxing songs)—see Materials and Methods section—the actual normalized stride length values were compared.

Overall, the results of this analysis show that our model can predict the kinematic responses to songs. In the following paragraphs we discuss in more detail (i) the audio feature extraction, (ii) the regression model, and (iii) the statistical analysis.

#### Audio feature extraction

First, a frame-by-frame audio analysis was done on both pitch and loudness (total loudness and separate loudness in six frequency sub-bands). Second, these loudness and pitch feature values were analyzed in each IBI, giving rise to beat-level feature vectors. In total 46 sonic beat-level features were extracted. Examples are features describing the onset of a beat (e.g. the position of the onset within an IBI), features summarizing the loudness in an IBI (mean, standard deviation, and center of gravity of the loudness samples), features defining the notes in an IBI (salience and pitch of the first, second, and third most salient notes per IBI), and features reporting cosine similarities between two subsequent IBI’s. Finally, for each of these 46 beat-level features the time pattern throughout the whole stimulus was analyzed, by checking evidences for increases or decreases of a feature value every two, three, four, or six beat periods. We refer to the study by Varewyck et al. [18] for more details on the audio feature extraction.

#### Regression model: training and features

Participants were instructed to walk in their own preferred tempo, which on average was 112.47 SPM (*SD* = 7.23). Of the 84 songs in our database 81 were actually played to the participants. Sixty of the 81 songs were played to five or more participants. One of those songs was an unfamiliar song with a complex rhythm. Because this song was quite different from the other songs and the averaged normalized stride length was based on only five values, we decided to leave this song out. Therefore 59 songs were used for training and testing a predictive regression model for normalized stride length. The root-mean-square error (RMSE) between the normalized measured stride length and predicted values is 0.27. The Pearson Correlation Coefficient (PCC) between the two is 0.75, meaning that 56% of the original variance in the measurements is explained by the model.

Three features occurred in all 10 cross-validation tests, indicating that these features were most important in affecting the stride length. Table 2 summarizes them. Feature 159 shows the strongest correlation with the normalized stride length, explaining 28% of the variance (PCC = −0.53). The feature is derived from an analysis of the note evidences measured in subsequent IBI’s. More precisely, this feature describes the frequencies (in chroma) of the third most salient pitch in each IBI. It has a high value if the spectral analysis of the temporal evolution of this feature reveals the clear presence of a frequency of one fourth of the beat rate, or once every period of four beats. The other two features are derived from an analysis of the individual loudness patterns in subsequent IBI’s, i.e. the loudness evolution in time of six different loudness sub-bands. Feature 60 describes the average loudness in sub-band two for each IBI. It has a high value if the spectral analysis of the temporal evolution of this feature reveals the clear presence of a frequency of one sixth of the beat rate, in other words once every six beats. Feature 98 reflects the variance of loudness in sub-band six for each IBI. It has a high value if the spectral analysis of the temporal evolution of this feature reveals the clear presence of a frequency of one third of the beat rate, or once every three beats.

**Table 2:** The most frequently selected sonic features (out of ten models) for stride length.

Id	Feature description	*μc*	*σc*	N	PCC
60	Evidence for a period of 6 beats in the average loudness in sub-band 2 in a beat period	−0.14	0.03	10	−0.47
98	Evidence for a period of 3 beats in the variance of the loudness in sub-band 6 in a beat period	−0.18	0.02	10	−0.50
159	Evidence for a period of 4 beats in the frequency (in chroma) in the third most salient note in a beat period (frequency = 0 if no third note is present)	−0.18	0.05	10	−0.53

For each feature we list the feature number (Id), the mean (*μc*) and standard deviation (*σc*) of the regression coefficients for these features in the models, the number of times (N) the feature was selected, and the Pearson Correlation Coefficient (PCC).

All three of the above features have negative regression coefficients, which means that high feature values are associated with relaxed walking, or the use of less vigor in the walking movement. Features 60 and 98 reveal loudness fluctuations with frequencies of one sixth and one third of the beat rate respectively. Analysis of the songs in our database reveals that such a ternary emphasis can either be found in songs with a ternary meter or by irregular patterns or longer melodies that divert attention from a binary emphasis in songs with a binary meter. The value of feature 159 is set to zero if only one or two notes are found in an IBI. Songs with the lowest values for this feature are songs with mainly drums and bass, like in hip-hop. The low values for this feature reveal the activating character of songs with little tonal diversity. This activating character could however be decreased by the presence of a more complex rhythmic structure, that weakens the binary emphasis.

#### Regression model: prediction

**Fig 2:**
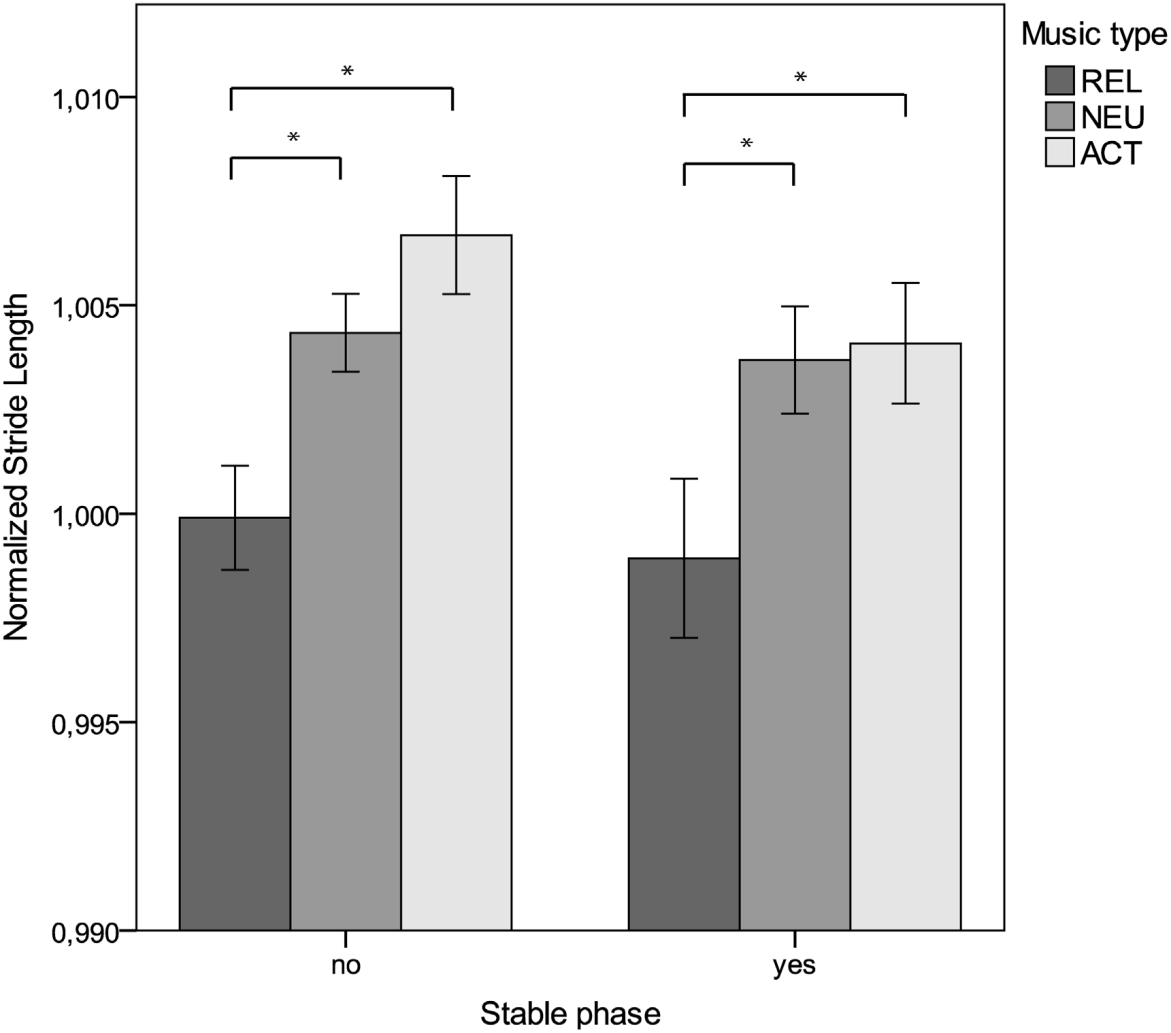
Normalized stride length for stable phase and non-stable phase walking. The figure depicts the normalized stride length values for different music types (REL, NEU, ACT) in stable phase trials and non-stable phase trials. A normalized stride length of value 1 represents an average stride length that equals the averaged stride length of walking in silence. Higher and lower values respectively indicate bigger or smaller stride length than in silence. Asterisks (*) indicate significance differences at *p* < .05.

Since the regression model was based on stride length values, we only explored the effect of music type (relaxing, neutral, activating) according to the model on actual stride length and not on velocity. In addition, the factor of walking phase coherently or not was studied. Apart from stride length being normally distributed, *D*(153) = 0.07, *p* = .07, the variances were equal for the six factor groups, *F*(5, 147) = 1.47, *p* = .20. Hence, an ANOVA with stride length being the dependent variable was performed, where music type and stable phase walking were used as fixed factors. There was a significant main effect of music type on stride length, *F*(2, 147) = 9.64, *p* < .001. There was no significant main effect of stable phase on stride length, *F*(1, 147) = 1.56, *p* = .21, and no significant interactions were found, *F*(2, 147) = 0.30, *p* = .74. Tuckey post hoc tests revealed that stride length was significantly larger for neutral music (*M* = 100.40%, *SE* = 0.09) than relaxing music (*M* = 99.94%, *SE* = 0.10), *p* = .004, for activating (*M* = 100.54%, *SE* = 0.10) compared to relaxing music, *p* < .001, but not for activating compared to neutral music, *p* = .60. Fig 2 clearly shows the difference between relaxing music and the other two types of music. Differences between neutral and activating music are smaller, even more so for walking with a stable phase than for walking phase incoherently.

### Kinematics and motivation

After the walking experiment participants were asked to rate the motivational qualities of the music they had just heard. To find out whether the motivational aspects of the music were related to performance, i.e. velocity, the BMRI-2 scores of the trials during which participants walked slower to music than to silence were compared to the trials where they walked faster to music than to silence. The differences between the two groups of BMRI-2 scores were normally distributed. Therefore, a dependent *t*-test was used to calculate the differences between the two groups. When participants walked faster than in silence they rated the music significantly higher with the BMRI-2 test (*M* = 25.39, *SE* = 0.82) than when they walked slower than in silence (*M* = 22.74, *SE* = 1.21), *t*(27) = −2.92, *p* = .01, *r* = .49.

We also explored a possible relationship between the motivational qualities of the music and the amount of phase coherence participants displayed while walking to music. The group of phase coherent trials was compared to the group of phase incoherent trials. Again, the differences between the two groups of BMRI-2 scores were normally distributed and a dependent *t*-test was performed. Walking in phase coherence with the music (*M* = 24.30, *SE* = 0.81) or not (*M* = 22.35, *SE* = 0.91) proved only to have a marginally significant influence on how the music was rated with the BMRI-2 test, *t*(28) = −1.95, *p* = .07, *r* = .35.

## Discussion

This study shows that musical expression has an effect on people’s walking velocity. Some music (called “activating”) increases velocity, whereas other music decreases velocity. Results also confirm that sonic features can predict the size and the direction of the velocity effect of a song. In addition, the study shows that phase-coherence has only a minor effect on walking velocity and finally, it shows that the velocity effect goes together with a motivational effect.

### Velocity effect of acoustic musical features on self-paced walking

Multiple studies underline the benefits of self-paced walking or exercise over moving at a prescribed tempo or intensity. A review by Williams [8] states that there is emerging evidence that self-paced exercise elicits more positive affective responses than prescribed intensity training, and that affective response leads to increased adherence to exercise programs. Williams pleads for a shift in physical activity guidelines, emphasizing performance of exercise at an intensity that ‘feels good’ rather than at a specific prescribed intensity. This could result in a more sustainable training experience and enhanced health outcomes. Similar findings have been reported in the field of gait rehabilitation. A study by Roerdink et al. [9] examined the use of different metronome rates in gait rehabilitation. They found superior auditory-motor coordination for pacing frequencies near the preferred cadence, suggesting that the efficacy of acoustic rhythms to influence gait degrades with pacing frequencies further away from one’s preferred cadence. In addition, a review by Nombela et al. [10] on rhythmic acoustic stimulation (RAS) for PD patients concludes that rhythms should be designed effectively, as they appear to lose therapeutic value when they are not tuned to the individual’s pace, or when they become more cognitively demanding. They encourage individually tailored future neurological music therapies for PD, attending to the specific clinical features and stimulus responding of the individual.

The background for this lies in the tight link between velocity (*v*), step frequency (*f*) and step length (*d*). Kuo [19] suggests that a person’s preferred *v*-*f* relation is based on minimizing an O_2_ consumption cost, and that this function can be captured by a mechanical model. As such, a given frequency has an effect on speed and therefore also on step length. Furthermore, Bertram and Ruina [20], who also studied this *v*-*f* relationship, concluded that there are differences in *v*-*f* relationships depending on which kinematic parameter is constrained. Other studies show the difference in variability on step size as a result from walking at different step frequencies. A study by Styns et al. [4] showed that walking at frequencies of 130 SPM or higher induced bigger differences in step size than walking with a lower cadence. In addition, Danion et al. [21] showed a clear effect of stride frequency on the stride length variability. When instructed to walk at different prescribed step frequencies and step sizes, both spatial and temporal variability appeared to be minimal at a frequency of 120 SPM compared to lower frequencies of 96 or 107 SPM, or higher frequencies of 137 or 151 SPM. Danion et al. [21] also propose a biomechanical explanation for longer steps having a higher consistency in step size: when taking large steps the joints get closer to their full flexion position, and muscles in the joints are stretched to their limits. Therefore, the larger the steps become, the less room there is to increase step size even further, thus decreasing irregularities of the stride. The above studies teach us to be careful with imposing a certain step frequency, especially if it is quite a bit higher or lower than a participant’s preferred cadence.

Regarding the feature selection process, we do not claim that the used 185 sonic features describe the acoustical space of the songs optimally. It is likely that other features are useful as well. Our basic thought simply was to use a broad range of features describing temporal fluctuations in energy and pitch. Although our regression analysis selected different sonic features than the one in the study by Leman et al. [5], results revealed that they represented similar characteristics in the music. In the current study as well as in the study by Leman et al. [5], the audio features showed that a change or emphasis every four beats has an activating, i.e. increasing effect on walking velocity and stride length. The most activating songs in our study were mainly 4/4-meter songs of the genre Disco, Hip-hop, or New wave. These songs had clear audible beats and in general chord changes every four beats. On the other hand, most of the relaxing songs lacked a clear audible beat and came from genres like Down-tempo, Soul, and Jazz. The relaxing effect on kinematics could be explained by an emphasis on ternary aspects of the meter, such as in songs with a 3/4-meter, or songs with syncopating melodies. These features seem to counteract the regular flow of a binary walking pattern.

Within the feature set many features represented similar characteristics, e.g. mean energy in an IBI for different frequency sub-bands. In the feature selection process as described by Leman et al. [5] only 10 of the 185 features are kept. The similarity between features resulted in quite similar feature values, which could easily have lead to other features coming out of the feature selection process. Another explanation for the selection of different features than in the study by Leman et al. [5], could be traced back to the music database. In the current study, no classical music was used, whereas 13% of the music was classical in the study by Leman et al. [5].

### Phase coherence and the velocity effect

Musical rhythm has been shown to have a stimulative effect on the human locomotion irrespective of any synchronization [22]. Using synchronous music while exercising, proved to have even more benefits, such as longer endurance [1], less perceived exertion [2], and lower limb discomfort [23]. In the current study, approximately half of the participants walked with a stable phase relative to the music, even without instruction. This is quite different from the results of Mendonça et al. [24] and Franĕk et al. [25], where almost none of the participants synchronized with the music without instruction. A study by Van Dyck et al. [26] offers an explanation for these differences. Runners were asked to run in their own tempo, while listening to music. First, the tempo of the music was matched exactly to the runner’s cadence and was subsequently increased or decreased up to a maximum of 3%. A tempo entrainment basin was found: participants spontaneously adapted their running tempo to the tempo of the music, up to tempo changes of approximately 2%. The fact that participants in our study were presented with music at a tempo that exactly matched their walking tempo, will notably have contributed to the high number of synchronized trials. The music tempo in the studies by Mendonça et al. [24] and Franĕk et al. [25] was matched more coarsely, augmenting the chance of having a tempo difference bigger than 2%, and thus making it harder for the music to have a subliminal effect on people’s walking tempo.

Even though no significant main effect of stable phase on stride length was found, the results of our study showed that differences in stride length between the types of music were smaller for phase coherent walking, than for phase incoherent walking. In other words, the velocity effect of music was bigger for phase incoherent walking than for phase coherent walking. The difference in effect size emphasizes the distinction between the process of phase incoherent walking and phase coherent walking. The *v*-*f* relation can explain this: velocity can only be changed by the stride length if the cadence remains stable. However, when a person does not walk in phase coherence with the music, velocity can be changed both by changes in stride length and changes in cadence. A higher or lower cadence could in turn have an impact on the stride length. This interdependence results in a bigger variance in walking behavior, and thus a bigger effect size on walking velocity and stride length.

How these differences in motor behavior are linked to the auditory or neural system is not clear. None of the participants were instructed to synchronize their steps with the music. This is nevertheless no guarantee that they were not aware of the music and some of them might have consciously adapted their walking to the music. The dynamic re-parameterization of motor behavior while walking to music is the result of continuous neuro-feedback, and can either be a conscious or an unconscious process. Stephan et al. [27] studied the influence of awareness on sensorimotor synchronization behavior. Results showed that movements could be adjusted both at a subconscious level and at a fully conscious level. However, only fully conscious motor control which involves motor planning, activated dorsolateral prefrontal cortex. Future research might reveal whether awareness can explain the differences in movement behavior: either walking phase incoherently or with a stable phase.

### Velocity effect and motivation

Our study revealed a significant relationship between the walking velocity and the motivational ratings (BMRI-2) [12]. Nevertheless, the relationship between walking in phase coherence and motivational scores was only marginally significant. The experimental setup with regard to measuring motivation has room for improvement. Instead of rating all the music after the walking experiment, the BMRI-2 scores might better reflect the motivational qualities of the songs when rated directly after hearing it during the exercise.

### Velocity effect size

In this study we were able to confirm the earlier findings of Leman et al. [5] on activating and relaxing music in ecological conditions (uninstructed self-paced walking with tempo-matched music). Nevertheless, the effect size on walking velocity, found in our study, was much smaller: up to 3% instead of up to 10%. Several explanations can be found for this discrepancy.

A first explanation concerns the difference in reference tempo. For most people, a cadence of 130 SPM is higher than their preferred self-paced cadence. According to the *v*-*f* relation, step sizes at self-paced tempo are larger, leaving less room to increase, whereas step sizes at higher tempi are smaller, thus leaving more room to increase. In other words, when step sizes are small the velocity effect of music can be larger.

A second explanation concerns the instruction. Only a small amount of studies have been dedicated to the effect of instructing people to synchronize, as opposed to spontaneous, or uninstructed synchronization. Two studies compared these differences in the field of social synchronization, more precisely in side-by-side walking. Van Ulzen et al. [28] explored the differences in amount of synchronization and phase locking, whereas Nessler and Gilliland [29] studied the kinematic differences between individual walking, uninstructed side-by-side walking, and instructed side-by-side walking. The latter study revealed that instructed synchronization may promote a more active control strategy. This was shown by the use of more, but smaller steps in order to actively adapt one’s walking pattern to another oscillating system. The results also showed an increase in the coefficient of variation for step size when instructed to synchronize, which could account for bigger effect sizes of music on step size when being instructed to synchronize to music. Additionally, instructed synchronization may have energetic considerations: healthy individuals adapt their stride length to their walking velocity to minimize energy expenditure [30], suggesting that for rehabilitative purposes instructed synchronization would be less desirable.

A third argumentation for the smaller velocity effect of music is a difference in the walking protocol. In the present study people were asked to keep walking for two blocks of 15 minutes, without being instructed to synchronize. Whenever a new song started playing, a participant was already walking at the same tempo as the song’s tempo. In the study by Leman et al. [5] participants had to stop walking after each song. Even though they were instructed to synchronize their walking to the music, the stopping in between songs could have had an impact on participants’ walking flow, resulting in bigger variances in step length. Another difference in experimental set-up concerns the reference stimulus. We compared the walking behavior of each song to the walking behavior on the 15 seconds of silence directly preceding each particular song. Leman et al. [5] compared the walking behavior of each song to the average walking behavior on six metronome trials that were evenly distributed over the course of the experiment. If a person was walking at 130 SPM for 15 minutes, the step length might have decreased over the course of these 15 minutes, while the cadence stayed stable. As a consequence, comparing step size to the average metronome walking behavior, actually comes down to comparing with an average step size at the middle of the experiment, which could result in a bigger variance of normalized step size.

### Relevance for gait rehabilitation

In the current study, an average increase in step length of 0.83% was found for walking on activating music. Since a decrease in step size is one of the major problems for Parkinson’s disease (PD) patients, the use of RAS in the form of activating music is an interesting line of research for rehabilitative purposes for this group of patients. De Bruin et al. [31] studied the use of music for walking rehabilitation with PD patients. They used highly familiar music for 30-minute walks, three times a week for a period of 13 weeks. A significant increase in stride length (0.70%) was found for patients with PD after the intervention period. An interesting path for future research might be to use activating familiar songs in a PD rehabilitation program, to try and increase stride length even more.

However, a study by Leow et al. [32] concludes differently. Step size while walking to high- and low-groove music did not increase compared to walking without music. This effect could have been caused by the actual instruction to synchronize to the beat. PD patients are generally seen as weak beat perceivers [32], as their beat perception is impaired by deficient basal ganglia function. Requiring PD patients to synchronize their steps to the beat increases attentional demand, which could worsen their gait. Our study was limited in the sense that it tested healthy subjects. Still, the fact that an adequate type of music has the capacity to elicit a spontaneous increase in walking velocity and stride length, holds great promise for PD patients. Automatically synchronizing to a beat when not instructed to could nevertheless be debatable [24, 25, 28, 29].

Another reason why Leow et al. [32] did not find an increase in stride length could be the type of music used in the experiment. We should be careful not to confuse high-groove music with activating music. High-groove music is said to “make you want to move”, to have danceable rhythms [33]. Stupacher et al. [34] demonstrated that high-groove music modulates the motor system activity. The modulations of the motor system in musicians are aligned with the beat during high-groove music. However, activating the motor system does not necessarily result in an increased step length. It could also result in an increase of vigor in vertical movements or movements in the upper body such as in dancing. An important element of groove according to Madison et al. [35] is syncopation: a disturbance in the regular flow of the rhythm, by placing accents where they would not normally occur. Such accentuation on the ‘off’ beat will however weaken a binary meter, which has been shown both in our study as in the study by Leman et al. [5] to cause music to have a relaxing effect on walking.

Although music can increase people’s step size, for PD patients we need to pay special attention to the type of music that is most suitable to do so and we need to question the additional load by instructing them to synchronize.

### Relevance in cyclic sports

Music has proven to positively influence athletes in all stages of exercise, from warm-up, to training and cool-down. Results from a study by Jarraya et al. [36] demonstrated positive effects of music with a tempo between 120 and 140 BPM during warm-up on high intensity performances. The power output during exercise was significantly higher when music was presented during the warm-up as opposed to having no music in the warm-up phase. It would be interesting to see in future research if the power output would increase if only activating music would be presented. Music or rhythmic stimuli also proved to be beneficial after intense exercise. Eliakim et al. [37] found that the use of popular music at 140 BPM during recovery significantly increases the activity level (measured by the number of steps), lowers absolute lactate levels, and augments the average decrease in RPE. The use of activating and/or high-groove music will probably be most equipped to stimulate an active recovery.

During training, music can be used in several ways to prevent injuries and regulate training. Relaxing music could for instance be used in running training programs: without decreasing the runner’s cadence, relaxing music could help runners to take smaller steps in order to prevent injuries caused by overstriding [38]. It could also be a tool in long distance running to enable to reduce heart rate, when it exceeds the anaerobic threshold, again without decreasing cadence. Other than preventing injuries, music can be used during training to enhance performance.

During a race it is not always permitted to use music. Nevertheless, the close link between our auditory and motor system shows promise to use auditory imagery as a way to improve our running performance during a race. A study by Meister et al. [39] revealed that simply imagining playing a song on the piano activates similar brain areas as when actually playing the song. In analogy, if an athlete mostly trains with a specific song or playlist, simply thinking about these songs during a race could activate the motor system in similar ways as by actually hearing the songs.

As for gait rehabilitation, our results emphasize the importance of using an adequate type of music while performing. If the aim of training is to increase velocity, preferably, activating music should be used. The 1–2% velocity increase as a result of listening to activating music might seem small. However, if this increase in velocity would also be achievable for running, it would lead to a significant one-minute-win for a top athlete running a marathon. Future research in this area could reveal the actual velocity effect of activating music for top athletes.

### Conclusions

Overall, our research question is highly relevant for the development of biofeedback systems in domains such as sports, rehabilitation, and healthy aging. Although uninstructed self-paced walking has multiple benefits, it is less likely to find a velocity effect of music, mainly because self-paced walking results in minimal variability in step size. Our study shows that the advantages of self-paced walking can go hand-in-hand with the spontaneous effect music has on walking velocity.

Furthermore, the current study proves the velocity effect of music for people that do not synchronize their step frequency with rhythmic acoustic stimuli, such as music. This opens up the possibility for using music for weak-beat perceivers, such as PD patients.

Finally, our study demonstrates a significant relationship between the motivational aspects of music and the velocity effect of music. Going towards a more individualized approach, this means that we can select music in advance that is both familiar and motivational for the participant in question, hence increasing the chances of music having an augmented effect on walking velocity.

## Supporting Information

S1 Table List of music that was selected and used in this study. The table contains the song ID, average BPM, artist and song title.
(XLSX)

S1 File Data used for the analysis. (XLSX)
